# Randomized controlled trial of stress management and resiliency training for depression (SMART-D)-pilot study

**DOI:** 10.1371/journal.pone.0328539

**Published:** 2025-08-19

**Authors:** Ashok Seshadri, Matthew Fuller-Tyszkiewicz, Laura Harper, Matthew M. Clark, Balwinder Singh, Sherry Chesak, Aparna S. Kaur, Jane McGillivray, Mark A. Frye

**Affiliations:** 1 Departments of Psychiatry & Psychology, Mayo Clinic, Rochester, Minnesota, United States of America; 2 School of Psychology, Deakin University, Geelong, Victoria, Australia; 3 Department of Nursing, Mayo Clinic, Rochester, Minnesota, United States of America; 4 Department of General Internal medicine, Mayo Clinic, Rochester, Minnesota, United States of America; Northwell Health Feinstein Institutes for Medical Research, UNITED STATES OF AMERICA

## Abstract

**Objective:**

Major Depressive Disorder (MDD) is characterized by high stress sensitivity and unsatisfactory response rates to standard treatments. Stress and depression share a bidirectional relationship. We, therefore, conducted a pilot randomized control trial (RCT) to understand if adjunctive stress management and resiliency training tailored for depression(SMART-D), can improve treatment outcomes in patients with MDD, receiving treatment as usual(TAU) with standard treatments (medications and/or psychotherapy), in real-world clinical settings, compared to a group receiving TAU.

**Methods:**

Participants with MDD, in a current depressive episode, were randomized to adjunctive SMART-D (delivered by video telehealth over 8 weeks), compared to TAU alone. Random assignment, blinding of raters and statistician were utilized. The primary outcome measure was baseline to end point change in depression [Hamilton Rating Scale for Depression (HAM-D] over a 6-month follow-up period using a mixed model regression analysis.

**Results:**

27 participants (mean age 47.9 ± 14 years, female 67%) enrolled in the study (TAU = 14, SMART-D + TAU = 13). Baseline mood ratings were in mild-moderate symptom severity (HAM-D)- SMART-D + TAU = 12.2 ± 6.6, TAU = 13.9 ± 5.7). Linear mixed model analysis showed significant Group*Time interaction for measures of depression (HAM-D) (B = 6.1 (CI = 1.5–10.8, P = .01) and perceived stress (PSS) (B = 5.5(0.5–10.6), p = .03) between the 2 groups at 3 months post follow-up ((HAMD)-SMART-D + TAU = 8.7 ± 4.3 Vs. TAU = 16.1 ± 6.3), but not at 6-months (SMART-D + TAU = 8.1 ± 5.4 Vs. TAU = 12.3 ± 5.5).

**Conclusions:**

A RCT of 27 adults with MDD provide initial support that an adjunctive resiliency intervention (SMART-D) for patients with MDD may positively impact symptoms of depression and perceived stress, earlier than standard care. A small sample size limits ability to draw firm conclusions. Further investigation is warranted, using larger samples.

Clinical Trials Registration I.D.# NCT04388748

## Introduction

Stress and major depressive disorder (MDD) share a well-established relationship. Acute and chronic accumulating effects of stress are associated with increased risk of the development, maintenance, and recurrence of MDD [1–4]. Depression may generate further interpersonal stress leading to chronic or recurrent depression [5–7]. Coping with stress involves the regulation of emotional, cognitive, and behavioral response to stress [8–10]. Successful adaptation (i.e., stress inoculation) can confer resilience to future stress [11]. Resilience is a construct defined as “the process of adapting well in the face of adversity, trauma, tragedy, threats or even significant sources of threat.” Developing resilience can help mitigate the negative psychological, biological, and social consequences of stress, through mechanisms of cognitive flexibility, emotional regulation, positive behavioral responses resulting in adaptive coping [12].

Evidence based psychotherapy interventions for MDD target depressive features related to motivational deficits and avoidance [Behavioral Activation (BA)] [13,14], negative automatic thoughts, self-schemas or core beliefs, cognitive errors, [Cognitive behavioral therapy (CBT)], [15] interpersonal relationship challenges and major life changes [Interpersonal therapy (IPT)], [16]. There is, however, a paucity of research studies investigating therapeutic effects of adjunctive stress management and resilience targeted interventions in clinical populations with MDD, who are receiving standard treatments with medications and psychotherapy. Interventions targeting resiliency through intentional, brief practices of gratitude, nonjudgemental attention, and maintaining an intentional mindset using principles of gratitude, compassion, acceptance, higher meaning and forgiveness, have been shown to improve mood, anxiety, perceived stress, and resilience in non-depressed populations [17–19].

In an earlier, single arm, open label feasibility study, in a sample of patients with mild to moderate MDD [20], an adjunctive 8-week Stress Management and Resiliency Training (SMART) program, showed evidence of significant improvement in symptoms of depression (p < .001), reduction in perceived stress (p = .002) and improvement in resilience (p = .03). The study also identified that participants had difficulties with practices of self-directed kindness, compassion, acceptance, and forgiveness [20].

Despite advancements in pharmacotherapy and psychotherapy, only 50–70% of patients with MDD achieve adequate response to standard treatments [21,22] Combining psychotherapy with medications only adds a small favorable effect compared to medication therapy alone [23,24]. Considering the prominent role of stress and the mediating role of resilience in translating stress to depression, we conducted this pilot RCT to provide preliminary directional data if adjunctive stress management and resiliency training, tailored for depression (SMART-D), can improve treatment outcomes in patients with MDD, who are receiving treatment as usual (TAU) (medications and/or psychotherapy) compared to a group receiving treatment as usual alone (TAU). The study hypothesized that the group receiving adjunctive SMART-D would experience improved depression outcomes in the current episode over a 6-month follow-up period, compared to TAU, by decreasing perceived stress and improving resilience over the 6-month follow-up period.

## Methods

The study protocol was approved by the Mayo Clinic Institutional Review Board and was registered in clinicaltrials.gov (NCT04388748). After providing written informed consent, participants were randomly assigned to receive 8 weeks (6 sessions) of adjunctive SMART-D to TAU or continue TAU with their current providers. The study was conducted between 18/12/2020 to 9/9/2022. Our study coincided with the COVID-19 pandemic, causing the need to modify the intervention delivery from planned in-person groups to virtual group sessions for all participants over secure HIPAA compliant audio video technology. Therefore, the entire study intervention was delivered virtually to all study participants. Funding for the study was limited to support from the department of psychiatry and psychology, Mayo Clinic, for research coordinator effort and remuneration for participants at the time of outcome measure completion.

### Inclusion criteria

Participants with MDD, in a moderate depressive episode, defined as Patient Health Questionnaire-9 (PHQ-9) [25] score between 10 and 19, who could speak English and provide written informed consent were eligible to participate in the study. Participants with co-morbid persistent depressive disorder and generalized anxiety disorder were included in the study.

### Exclusion criteria

Bipolar disorder, active psychosis, active suicidal ideations, and active substance abuse meeting criteria for substance use disorders except nicotine, obsessive compulsive disorder, active panic disorder with agoraphobia or other phobic disorder, active posttraumatic stress disorder, active severe personality disorders were excluded. Patients experiencing a severe major depressive episode and pregnant women were excluded.

### Procedure

Once enrolled, participants completed the Structured Clinical Interview for Diagnosis version 5 (SCID) modules A, D, E, F [26] to confirm the diagnosis of MDD. Clinical and demographic characteristics obtained during this study visit included age of onset of depression, number of previous lifetime episodes, duration of current or recent depressive episode, current medications, and any current psychotherapy type. Baseline clinical measures were obtained for symptoms of depression, perceived stress level, resilience, quality of life, current burden of stress(using Holmes and Rahe Current burden of stress scale) [27], and adverse childhood experiences (Table 1).

**Table 1:** Content of six SMART-D sessions.

Agenda	Insight	Practices
Week 1	Introduction to Stress - Acute and Chronic - The Good, Bad and the Ugly - The Stressed Brain - Fear, Fatigue and Focus - Default Mode vs Focused Mode What’s the SMART Approach? Train attention to the present moment with brief, intentional practices in the focussed mode Maintain a resilient mindset to face stress	Morning gratitude practice “Before you get out of bed, think of 5 people in your life, that you are thankful for. Send them a silent gratitude”- 2 minutes
Week 2	Mindful Presence Train your attention to observe the present moment Attention to the people in your life Attention to your surroundings	Two-minute rule: Nonjudgmental attention towards a loved one Kind Attention: Silent “I wish you well” Curious Moments: Find details within commonly encountered stimuli Kindness to Self
Week 3	Resilient Mindset I Training Phase: Monday-Gratitude Tuesday- Compassion Wednesday- Acceptance Thursday- Higher Meaning Friday- Forgiveness	Gratitude - Lower the gratitude threshold Compassion to others and self - Kindness + Action = Compassion
Week 4	Resilient Mindset II Training Phase: Monday-Gratitude Tuesday- Compassion Wednesday- Acceptance Thursday- Higher Meaning Friday- Forgiveness	Meaning - Agent of Love and Service Acceptance - Of people and situations Forgiveness - Choose to let go of anger
Week 6 and Week 8	SMART- Integrated approach	Develop individual ideas for integration into lifestyle

### Follow-up assessments

The first follow-up assessment occurred at the end of the 8-week SMART-D for both the comparison arms. The subsequent follow-ups occurred at three- and six-months post- SMART-D. Measurements were conducted by telephone, or electronically through the web-based patient portal. Participants were remunerated $20.00 in the form of a Mayo Clinic check for completing outcome measures at each assessment time point of the study. All the outcome measures were self-rated instruments, except the Hamilton rating scale for depression – 17 item (HAMD), that was delivered by one of two trained, independent and blind raters over the phone.

### Randomization and blinding

Randomization sequence was generated using Microsoft Excel 2013 using random block sizes of four random numbers by an independent researcher not involved with the study. Allocation sequence was concealed from the research coordinator assessing participants, using sequentially numbered, opaque, sealed, and stapled envelopes. Due to the nature of the study design, blinding of participants was not possible after randomization. Outcome assessors were blind to the group allocation. Study participants were informed not to share their group condition to the study personnel. The SMART-D intervention was delivered by the same researchers (A.S and S.C.) for all the intervention groups. Statistical analysis was performed (A.S.) maintaining the blind and broken only after all analysis were completed and checked by a second senior study researcher (M.F.T).

### Intervention

SMART is a resiliency training program created at the Mayo Clinic, based on the theoretical foundations of Attention & Interpretation Therapy (AIT) [28–30]. Key components of SMART include identifying emotional and cognitive signs of stress (e.g., loss of focus, experience of fatigue, increase in fear, and rumination), while recognizing different thinking modes of the brain- the default mode (where passive daydreaming and ruminative activities occur) and the focused mode (where brief intentional activities are performed). Attention training emphasizes mindful presence with practice of brief, externally focused, intentional activities in the focused mode, that reinforce the positive relationship with the external world through experiencing positive emotions. These include brief practices of morning gratitude to set the tone for the day (“As soon as you wake up, think of 5 people you are thankful for”), finding novelty in close relationships (2-minute rule-“Meet your family when you come home, as if you were away for a month, with undivided attention, and delay judgement for 2 minutes”), using moments of kindness (1 minute- Silent “I wish you well” when you encounter strangers or before meeting someone”), and using curiosity to experience the physical world (“Take some time to observe the details in things you encounter every day”). The cognitive aspect of training seeks to maintain a resilient mindset to reinterpret stressful life events using the principles of gratitude (ex. “thankful for what’s right”), compassion (ex. “kind action towards self and others”), Acceptance (ex. “accepting what went wrong while working to make things better”), Higher meaning (ex. “what can I learn from this?”) and Forgiveness (ex. “I choose to forgive to let go of anger). SMART involves delivering the intervention in a structured format, usually in a group setting that allow peer to peer and participant-provider interactions. The core concepts are introduced within the first 4 weeks, with follow-up sessions designed to develop individual ideas, identify barriers or difficulties in practice to find potential solutions.

The SMART-D intervention emphasized the principles of self-directed kindness, compassion, acceptance, and forgiveness, to specifically target negative self-concepts of depression (Table 1). It comprised 6 group sessions delivered over 8 weeks, with minimum 4 to maximum 8 participants per session. The intervention was delivered by 2 study therapists, who were trained and certified by the program creator, Dr. Sood, with one lead therapist (study PI, A.S.) and one co-therapist (Co-I, S.C.) for all the group sessions. The sessions utilized a HIPPA compliant real-time audio-visual connection via Zoom video through the Mayo Clinic Patient Portal with the first four sessions occurring weekly, and sessions five and six, occurring on week 6, and week 8 respectively. Each session was of 90-minute duration. The sessions included a brief introduction and review of home practices, followed by a presentation, based on the book “Mayo Clinic Handbook for Happiness” [30] accompanied by group discussion of each topic. All participants, randomized to the intervention group, received a copy of the book at no cost. Although structured, the intervention is not manualized. Therefore, to enhance treatment fidelity, the same PowerPoint slides were used for all sessions and random sessions were audio recorded to be reviewed by a content expert. SMART-D participants who completed 3 out of 6 sessions were considered study completers.

### Treatment as usual

Considering the study was conducted in real world clinical settings, all participants, in both groups, continued their routine mental health care with their outpatient treatment providers, including medication management and psychotherapy. Treatment providers for all participants were allowed to make any clinically necessary treatment changes and were not asked to modify any aspect of care they provided during study participation. The research team contacted the participants from both groups at 3 months and 6 months after the initial 8 week intervention period. Participants from the TAU group were offered the chance to participate in the SMART-D intervention at the conclusion of the six-month follow-up period. However, no outcome measurements were recorded.

### Outcomes

Primary and Secondary Outcome Measures:

Depression, assessed by the Hamilton rating scale for depression – 17 item (HAMD) [31] was the primary outcome measure. Remission was defined as scores of ≤ 7 [32]. Secondary outcome measures were perceived stress, resilience, and overall quality of life. Perceived stress was measured with the Perceived Stress Scale (PSS), a 10-item self-administered scale, measuring the degree to which one’s life is appraised as stressful. Scores of 20 or higher are associated with high perceived stress [33]. Resilience was measured using the Connor-Davidson Resilience Scale (CD-RISC), a 25 item self-rated scale, each item rated from 1 to 4. Higher scores are associated with greater resilience. Mean scores of 80.4 ± 12.8, was obtained from a random national sample of US adults in one study [34]. In comparison, lower scores are reported in outpatient depressive samples (57.1 ± 13.3) [34]. Quality of Life (QOL) was measured using a 6-item, 0 (as bad as it can be) to 10 (as good as it can be) linear analogue self-assessment (LASA) scale that inquiries about overall, social, emotional, spiritual, mental and physical quality of life [35]. Previous studies of MDD have used the LASA to measure QOL [36]. Overall QOL scores were identified a priori as the QOL domain of interest for this study.

### Power calculation

The sample size required to detect statistically significant differences between the 2 groups, assuming a moderate standardized mean difference of 0.7, alpha level = 0.05 (two-tailed), and power of 80%, would be 161 participants in each group. Given that we were aiming to conduct a pilot study, we aimed to enroll 80 participants between the 2 groups, however, limited funding and time resulted in a smaller, underpowered sample. Considering our statistical plan for this pilot study involved analytical modeling, we aimed for a larger sample to produce more reliable results, despite being a pilot intervention. We were also concerned about potential for COVID-19 pandemic related dropout rates, in aiming for a larger sample for a pilot study.

### Statistical analysis

Statistical analysis was carried out using SPSS software (Version 29). The SMART-D and TAU groups were compared on baseline demographic and clinical characteristics. Missing data were addressed using conditional maximum likelihood. Internal consistency was measured using Cronbach’s alpha for each measure, at each time point, for each group. Tests of normality were checked using normality tests (Kolmogorov-Smirnov and Shapiro-Wilk test) and Q-Q plots at each time point for each group. Primary analysis applied intention-to-treat analysis, i.e., as randomized regardless of intervention received, to compare treatment outcomes of depression, with participants analyzed based on their scores at baseline, post-SMART-D intervention, 3-month and 6-month follow-up time points between the two groups. Results are presented as mean differences (and 95% CIs), with p-values. Results will be reported in accordance to CONSORT 2010 guidelines [37].

Analysis of the primary outcome (depression) and secondary outcomes (perceived stress, resilience) were conducted using a linear mixed effects regression model, with group allocation (2 levels) and time of visits (4 levels), being independent variables with HAMD scores being the primary dependent variable. The analysis was conducted at the level of group (SMART-D + TAU vs. TAU), time (End of intervention, 3 months, and 6 months post-intervention), and group X time respectively, with the group X time interaction being the main level of interest. For each outcome, a single mixed effects regression model was fitted incorporating baseline, post-intervention, 3- and 6-month data using random effects to allow for the repeated measures within an individual. Significance was tested at p < .05 (two-tailed) for the primary analysis. Considering the small sample size, we could not complete secondary regression analysis to study the effects of co-variates or perform moderator analysis. The data analysis plan was finalized prior to final data lock.

## Results

**Fig 1:**
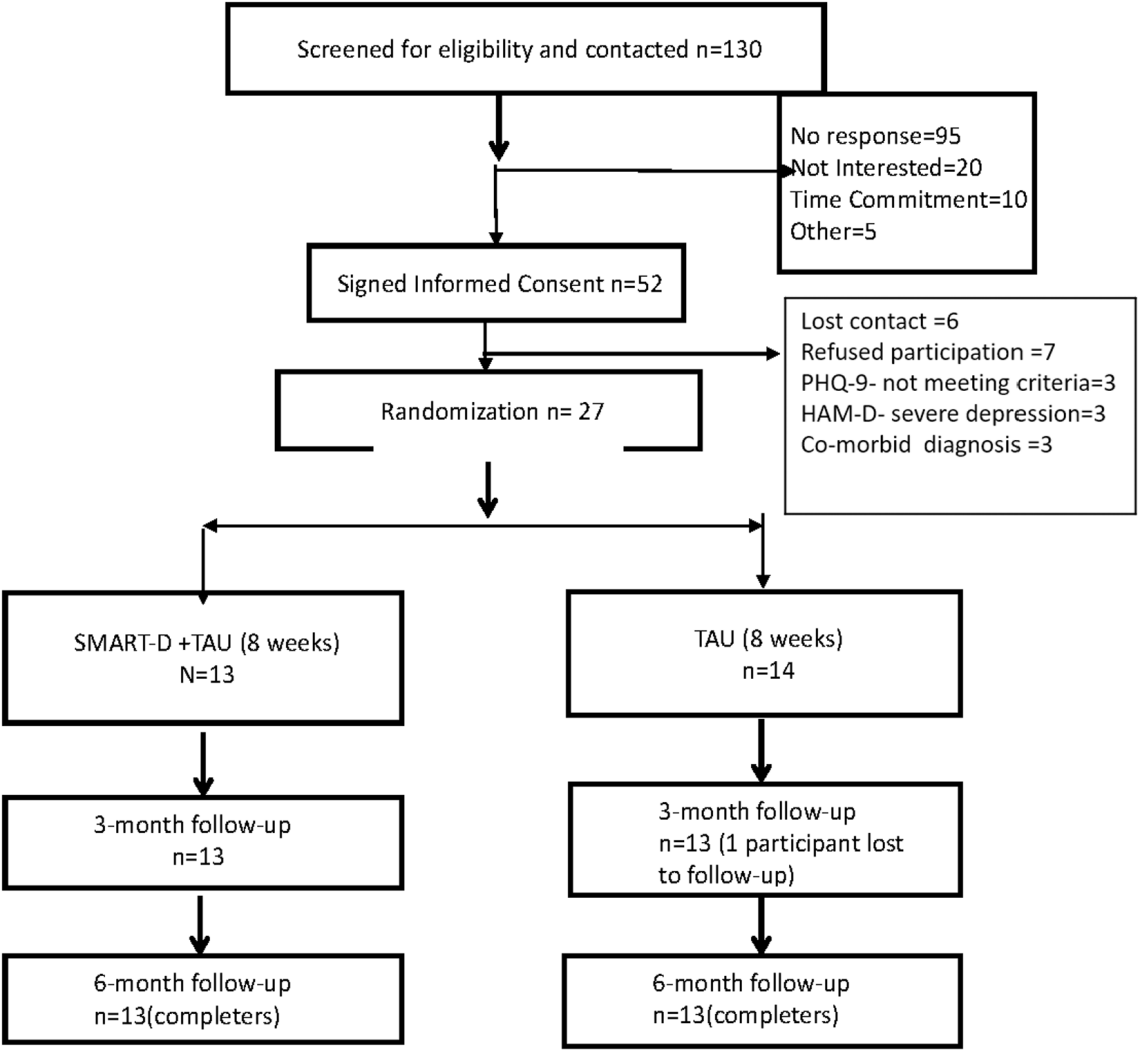
Consort Flow Diagram for SMART-D + TAU Vs. TAU Pilot RCT.

Total of 182 potential participants were contacted. Among people who responded (n = 87 (48%)), Fifty-two (52) participants signed informed consent (60% enrollment). Reasons for not participating included, “Not Interested” = 20 (21%), “Time Commitment=10 (10%), “Other” = 5 (5%). Twenty-seven (27) participants entered the randomization phase of the study. Fourteen (14) participants were randomized to the TAU group while thirteen (13) participants entered the SMART-D + TAU group. Twenty-six participants completed the clinical trial (96% completion rate). One participant dropped out at the three-month follow-up point from Group 1(Fig 1). No major adverse events occurred during the clinical trial. Twenty-six out of twenty-seven participants were receiving medications (SMART-D + TAU = 100%, TAU + 92%). Fifteen study participants (TAU = 7(50%), SMART-D + TAU = 8(61%)) were receiving concurrent psychotherapy.

Missing data analysis showed 6.6% missing data, the pattern of missingness consistent with missing completely at random. (Little’s MCAR test-Chi-Square = 21.4, df = 21, P = 0.43) Internal consistency (Cronbach’s alpha) estimates for the primary outcome measure (HAM-D), ranged between 0.76–0.84, across the two groups for six of eight measurements. Internal consistency estimates for CD-RISC (0.86–0.96), and PSS (0.79–0.92) across the multiple time periods did not reveal concerns about reliability.

Baseline demographic characteristics are presented in Table 2. Majority of the study participants were female (n = 18, 66%). Considering the small sample size, it is possible that baseline characteristics between the 2 groups may not have been evenly distributed between the 2 groups.

**Table 2:** Baseline Characteristics.

	Treatment As Usual (TAU) n = 14	SMART-D+ Treatment As Usual (TAU) n = 13
Age	50.1 ± 14.8	45.5 ± 12.9
Sex (%Female)	57%(n = 8)	77%(n = 10)
Education	4-year College- 57% High School or less- 14%	4-year College-69% High School or less- 8%
Employment	9(64.3%)	10(76.9%)
Married/Partnered	43%(n = 6)	62%(n = 8)
Age of 1^st^ depressive episode	25.2 ± 11.0	24.8 ± 13.1
Lifetime Hospitalizations	5(35.7%)	3(23.1%)
ECT	1(7%)	0
TMS	1(7%)	0
Ketamine	2 (14.2%)	0
Current Psychotherapy	7 (50%)	8(61%)
Medications		
SSRI/SNRI	13	10
Bupropion	7	6
2 or more Medications	9	9
No medications	1	1
Adverse Childhood Experiences	3.2 ± 2.2	3.00 ± 2.6
Current Burden of Stress (Holmes Rahe scale)	222.7 ± 148.3	208.00 ± 100.4
Caregiver Stress	6 (43%)	7 (54%)
Relationship Stress	8 (57%)	9 (69%)
Work Stress	8 (57%)	9 (69%)
Housing stress	4 (29%)	1 (8%)
Financial stress	7 (50%)	7 (54%)
Baseline HAM-D*	13.9 ± 5.7 CI = 10.5–16.7	12.2 ± 6.6 CI = 7.8–15.5
Baseline PHQ9*	12.9 ± 4.3 CI = 10.1–14.6	12.6 ± 4.9 CI = 9.6–15.6
Baseline CD-RISC*	56.7 ± 11.5 CI = 50.5–64.2	55.0 ± 20.9 CI = 43.2–67.8
Baseline PSS*	21.4 ± 5.8 CI = 18.3–24.9	24.5 ± 7.8 CI = 19.8–29.1

*Baseline continuous variables presented with standard deviation and 95% confidence intervals (CI).

**Fig 2:**
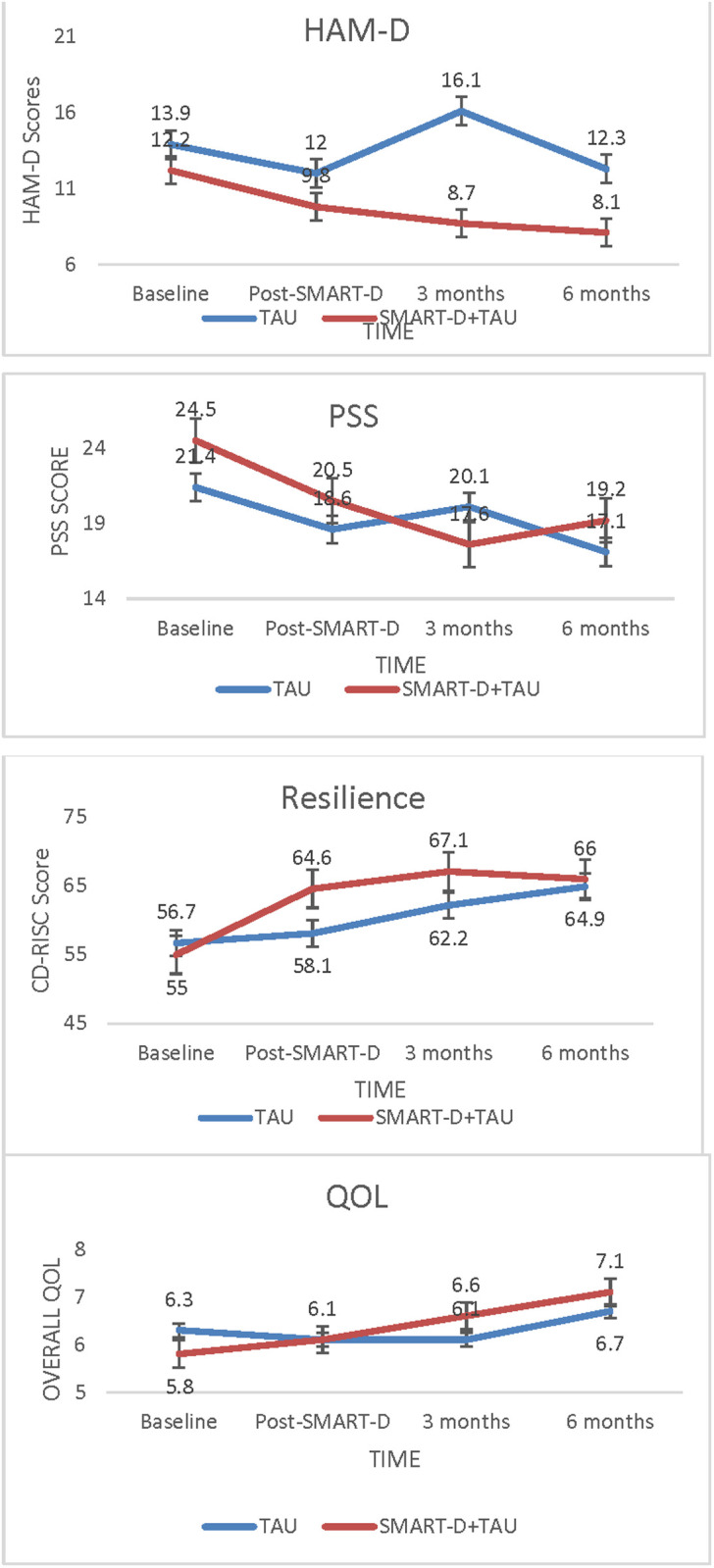
Depression, Perceived Stress, Resilience and Overall QOL from baseline, Post-SMART-D, 3-months and 6-months for SMART-D + TAU Vs. TAU. HAM-D- Hamilton Rating Scale for Depression-17. PSS- Perceived Stress Scale. CD-RISC- Connor-Davidson Rating Scale for Resilience. QOL- LASA Overall QOL.

The primary outcome measure for depression (HAM-D) (Table 3) showed progressive decrease from baseline (12.16 ± 6.6), to 3 months (8.69 ± 4.34) and 6 months (8.09 ± 5.4), in the SMART-D + TAU group compared to TAU (baseline = 13.98 ± 5.7, 3-months = 16.14 ± 6.3, 6-months = 12.29 ± 5.5) (Fig 2). The SMART-D + TAU group also showed a greater decrease in PSS scores at the 3-month follow-up period (baseline = 24.5 ± 7.8, 3-months = 18.1 ± 5.6, 6-months = 19.5 ± 4.3) compared to the TAU group (baseline = 21.6 ± 5.7, 3-months = 20.1 ± 5.9, 6-months = 18.3 ± 6.8).

**Table 3:** Primary Outcome Measure- HAM-D (Mean ± SD).

Time	TAU	SMART+TAU
Baseline	13.98 ± 5.7	12.16 ± 6.6
Post Smart	12.00 ± 7.1	9.78 ± 8.0
3 months	16.14 ± 6.3	8.69 ± 4.34
6 months	12.29 ± 5.5	8.09 ± 5.4

CD-RISC showed a greater increase in resiliency scores, immediately following the intervention compared to TAU, which was maintained over the following 6 months(baseline = 55.5 ± 20.9, Post-SMART-D(8 weeks)=64.6 ± 15.9, 3-months = 67.1 ± 10.0, 6-months = 66.0 ± 13.9), in contrast to the TAU group, where the improvements occurred more slowly, over 6 months(baseline = 57.4 ± 11.9, Post-SMART-D(8 weeks)=58.1 ± 18.3, 3-months = 62.7 ± 11, 6-months = 63.4 ± 16.8)(Fig 2).

SMART-D + TAU group showed a gradual improvement in their overall QOL (baseline = 5.8 ± 2.2, 3-months = 6.6 ± 1.9, 6-months = 7.1 ± 1.6), compared to the TAU group (baseline = 6.3 ± 1.4, 3-months = 6.1 ± 1.6, 6 months = 6.7 ± 2.1), who reported minimal change over the study duration.

Linear mixed model regression analysis showed a statistically significant group X time interaction between baseline (before SMART-D) and 3 month follow up, after SMART-D, for depression, [HAM-D (B = 5.6(0.8–10.4), p = 0.02)] and perceived stress, [PSS, (B = 5.5(0.5–10.6), p = 0.03)] where the SMART-D + TAU group, showed a significantly greater reduction in depression and perceived stress scores at the 3 month follow-up point from baseline, compared to TAU. The group X time interaction for depression between baseline and six month follow up period, however, did not reach statistical significance (B = 2.4(−2.4–7.1), p = 0.33), although the SMART-D + TAU achieved a greater mean reduction (MD = 4.1, SD = 2.66) in HAM-D from baseline, compared to TAU (MD = 1.69, SD = 0.95) (Table 4). However, considering the small sample size, the results from analytical modeling need to be interpreted cautiously.

**Table 4:** Results of SMART-D + TAU Vs. TAU for primary and secondary outcome measures after intervention, 3-month and 6-month follow-up (presented as mean difference (MD) with 95% Confidence intervals (CI), Cohen’s d and p-value.

	HAMD-17			Connor-Davidson-Resilience		
Predictor	MD (95% CIs)	Cohen’s d	p	MD (95% CIs)	Cohen’s d	p
Time * Group						
Baseline vs Post SMART-D	0.4(−4.7-4.5)	0.06	.97	−8.2(−7.7--.3.)	0.49	.15
Baseline vs 3-month FU	5.6(0.8-10.4)	0.91	.02*	−6.6(−17.6- 4.6)	0.39	.24
Baseline vs 6-month FU	2.4(−2.4-7.1)	0.39	.32	−2.8(−14.0-8.4)	0.16	.62
	**PSS-Perceived Stress**					
Predictor	MD (95% CIs)	Cohen’s d	p			
Time * Group						
Baseline vs Post * Group	1.1 (−4.0-6.1)	0.17	.68			
Baseline vs 3-month FU	5.5(0.49-10.6)	0.82	.03*			
Baseline vs 6-month FU	1.5(−3.45-6.5)	0.23	.55			

* Denotes Statistical difference.

At the 3 month and 6-month follow-up periods, 31%(n = 4/13) and 54% (n = 7/13) of SMART-D + TAU participants, respectively, were in remission, defined by HAM-D ≤ 7. The TAU group, in comparison, had 0% (n = 0/14) and 28%(n = 4/14) in remission at the same time points, respectively. This suggests that a greater proportion of SMART-D + TAU group participants, showed evidence of sustained recovery, while the TAU group showed a greater tendency for relapse. The SMART-D group had a greater and earlier mean increase in resilience over the study period, compared to TAU, that peaked by the 3-month follow-up period, however, this did not reach statistical significance within our time X group model.

Treatment effects (Cohen’s d) for depression, calculated based on differences between baseline to each timepoint respectively, show no difference at the end of the SMART-D intervention (Cohen’s d = 0.06), however a large effect size of 0.91 was found at 3 months and a moderate effect size of 0.39 at 6 months, in favor of the SMART-D + TAU group compared to TAU. Perceived stress showed small effects post intervention but large effects at 3 months (Cohen’s d = 0.82) and moderate effects at 6 months (Cohen’s d = 0.55) respectively. Resilience showed moderate effects, immediately post intervention in the SMART-D + TAU group compared to TAU (Cohen’s d = 0.48). Effects for Resilience reduced slightly to 0.39 at 3 months and further to 0.16 at 6 months.

## Discussion

This study reports the results of a pilot RCT of adjunctive stress management and resiliency program for MDD, conducted in real world settings. The results showed that the SMART-D intervention, augmented to TAU, was associated with a statistically significant and earlier reduction in depression at 3 months post intervention compared to TAU alone. The intervention group continued to sustain improvement over a 6-month follow-up period. Concurrently, a statistically significant improvement in perceived stress was observed over the same period. However, there was no statistically significant difference between the 2 groups for any of the outcome measures, at the 6-month follow-up period.

The SMART-D + TAU group had a greater and earlier mean increase in resilience over the study period, compared to TAU, which peaked immediately post-intervention, and moderated slightly by the 3-month follow-up period. However, change in resilience did not achieve statistical significance at the time X group level of the model. The improvement in depression and perceived stress followed the improvement in resilience and occurred at the 3 month and the 6-month follow-up periods.

This may suggest that the real benefits for depression occurred over the longer term, with improving resilience facilitating the subsequent improvement in perceived stress and depression in the intervention group. Depression and resiliency have been shown to be negatively correlated [34]. This may explain why it was only at the 6-month follow-up timepoint that the TAU group showed improvement in resilience, which coincided with slightly improved depression. This observation raises the question whether improvement in resilience in the SMART-D group was a direct outcome of the intervention and if improvement in resilience is a necessary precursor for improving perceived stress and depression. A larger sample is required to show if the intervention itself produces an improvement in resilience, in comparison to an active control condition.

The lack of statistical significance at the 6-month follow-up period could be attributed to the small sample lacking adequate power to detect significant differences over an 8-month period, resulting in a Type II error. Additionally, floor and ceiling effects may have impacted the results. For example, the SMART-D group had a mean HAM-D score of 12.2 at baseline and a mean score of 8.1 at 6-month follow-up, creating the possibility of a floor effect for further improvement on the HAM-D [19].

SMART-D shares characteristics with other psychological therapies, with its emphasis on emotional recognition and staying in the present moment (mindfulness), focus on eudemonic practices (Positive Psychology) and acceptance principles (Acceptance and Commitment Therapy (ACT)) Mindfulness based cognitive therapy (MBCT) and ACT, have shown positive evidence of small to moderate treatment effects for MDD, while Positive psychotherapy was comparable to CBT [38] In contrast to these techniques, SMART emphasizes resilience to stress as the main target, using regular practices of focused attention, with brief, intentional, externally directed activities. Instead of a nonjudgmental stance, SMART emphasizes a mindset of gratitude, compassion, acceptance, higher meaning and forgiveness to interpret life experiences and stressors [39].

Regarding what may have helped participants in the SMART-D group, they could have learned to recognize negative emotional and ruminative states, leading to positive coping with brief, externally focused practices, thereby helping reduce rumination. Practices of gratitude and compassion could have led to improved interpersonal relationships, leading to reinforcement of social supports, and reducing stress generation. Intentional practice of gratitude could have helped improve feelings of isolation. Practices of self-compassion, self-acceptance, and self-forgiveness could have helped reduce negative self-esteem, feelings of guilt and improve feelings of self-adequacy.

The strength of our pilot study is the high external validity, given it was conducted in real world settings with established patients with long standing history of major depression, diagnosed using standardized methods. The study highlights the adjunctive benefits of directly addressing stress and resilience in addition to standard treatments of pharmacotherapy and psychotherapy for depression.

## Limitations

Our study has several limitations. A small sample size precludes making firm conclusions, as risk of both type 1 and type 2 error are high. Additionally, fitting analytical models to small sample sizes may be associated with bias, hence conclusions need to take this into account. Despite randomization, it is possible that the TAU group may have had greater current depression severity in comparison to the SMART-D + TAU group. Lack of an active control group could also have led to overestimation error. We were not powered to perform secondary analysis, to understand the relationship between psychosocial variables, illness severity, impact of COVID-19, medication therapy, type and dose of psychotherapy, and treatment outcomes. Considering our study was a therapeutic intervention compared to TAU, blinding of participants was not possible after randomization. The fact that the same therapists conducted all the group interventions could be a strength, however it increases the risk of performance bias. The therapists were not blind to the study hypothesis. A strong placebo effect could have contributed to positive response in the intervention group, and dejection in the TAU group. However, the finding of small effects for depression, at the end of the intervention period, suggests reduced influence of the placebo effect. Finally, the participants were of high educational background, and while we did not record ethnicity, majority of participants were white, so how these finding apply to more diverse or underserved populations is unknown.

## Future directions

This pilot study provides preliminary data that an adjunctive resiliency intervention may benefit depressive patients in producing an earlier and more sustained treatment response for depression, compared to usual treatment alone over a 6-month follow-up period. Larger randomized controlled trials, which include an active control group, should focus on the role of early intervention with resiliency training to confirm these results. It is important to understand if early resiliency training can accelerate the treatment response to standard treatments and reduce recurrence, considering the prominent role of stress in causing relapse and remission of depression.

## Conclusions

A pilot randomized controlled trial of an adjunctive stress management and resiliency training program, tailored for depression, provides initial support for improving depression, including an earlier and sustained treatment response, over six-month follow-up, in comparison to treatment as usual. However, we cannot draw any firm conclusions, due to a small sample. The mechanisms of change could be related to improved resilience and decreased perceived stress following the intervention. Larger, adequately powered, randomized controlled trials, are needed to further explore the relationship between targeted stress and resiliency interventions in improving treatment outcomes for MDD.

## Supporting information

S1 File Raw scores. (DOCX)

S2 File Consort Checklist. (DOCX)

S3 File Protocol. (DOCX)
