# Supplementary material for: Randomized controlled trial of stress management and resiliency training for depression (SMART-D)-pilot study
Source: PLoS One. 2025 Aug 19;20(8):e0328539. doi: 10.1371/journal.pone.0328539 (PMC12364347; doi:10.1371/journal.pone.0328539)
Supplement: S1 File — (DOCX) [file pone.0328539.s001.docx]

**Supplement: 1:**

**Raw Scores: PHQ-9:**

| Time | TAU (Mean±SD) | SMARTD+TAU (Mean±SD) |
| --- | --- | --- |
| Baseline | 12.9 ±4.3 | 12.6 ±4.9 |
| Post Smart | 10.4 ± 6.0 | 9.7 ± 4.7 |
| 3 months | 10.5 ± 5.0 | 8.7 ± 4.0 |
| 6 months | 11.1 ± 4.9 | 8.5± 3.8 |

**Raw Scores- Connor Davidson Resilience Scale:**

| Time | TAU (Mean±SD) | SMARTD+TAU(Mean±SD) |
| --- | --- | --- |
| Baseline | 56.7 ± 11.5 | 55.0 ± 20.9 |
| Post Smart | 58.1 ± 18.32 | 64.69 ± 15.9 |
| 3 months | 62.2 ± 11.00 | 67.1 ± 10.0 |
| 6 months | 64.9 ± 17.0 | 66.0 ± 13.9 |

**Raw Scores: Perceived Stress Scale**

| Time | TAU(Mean±SD) | SMARTD+TAU(Mean±SD) |
| --- | --- | --- |
| Baseline | 21.4 ± 5.8 | 24.5 ± 7.8 |
| Post Smart | 18.6 ± 6.3 | 20.7 ± 6.2 |
| 3 months | 20.1 ± 5.9 | 17.6 ± 5.6 |
| 6 months | 17.7 ± 6.8 | 19.2± 4.3 |

**QOL- Overall QOL**

| Visit Time | Overall QOL |  |  |  |
| --- | --- | --- | --- | --- |
|  | TAU -Mean | SD | SMARTD+TAU-Mean | SD |
| 1.00 | 6.29 | 1.44 | 5.85 | 2.19 |
| 2.00 | 6.15 | 2.23 | 6.17 | 2.33 |
| 3.00 | 6.14 | 1.56 | 6.58 | 1.93 |
| 4.00 | 6.67 | 2.15 | 7.10 | 1.60 |
