# Supplementary material for: Randomized controlled trial of stress management and resiliency training for depression (SMART-D)-pilot study
Source: PLoS One. 2025 Aug 19;20(8):e0328539. doi: 10.1371/journal.pone.0328539 (PMC12364347; doi:10.1371/journal.pone.0328539)
Supplement: S3 File — (DOCX) [file pone.0328539.s003.docx]

| **Protocol Template** |
| --- |

**Note: If this study establishes a human specimen repository (biobank) for research purposes, do not use this template. Use the Mayo Clinic Human Specimen Repository Protocol Template found on the IRB home page under Forms and Procedures at http://intranet.mayo.edu/charlie/irb/**

**First-time Use:** Use this template to describe your study for a new IRB submission.

1. Complete the questions that apply to your study.
2. Save an electronic copy of this protocol for future revisions.
3. When completing your IRBe application, you will be asked to upload this document to the protocol section.

**Modification:** To modify this document after your study has been approved:

1. Open your study in IRBe. Click on the study ‘Documents’ tab and select the most recent version of the protocol. Save it to your files.
2. Open the saved document and activate “Track Changes”.
3. Revise the protocol template to reflect the modification points , save the template to your files
4. Create an IRBe Modification for the study and upload the revised protocol template.

| **General Study Information** |
| --- |

Principal Investigator: Ashok Seshadri, M.D.

Co-Principal Investigator: Mark A. Frye, M.D.

Co-Investigators: Matthew M. Clark, Ph.D., L.P.; Sherry Chesak, Ph. D.; Sarah Stinson, M.S.

Collaborators: Amit Sood, M.D. (advise on the SMART intervention); Jane Mcgillivray, Ph.D. (serve as mentor – will not receive study data); Matthew Fuller-Tyszkiewicz, Ph.D. (supervision with trial design, planning and statistical analysis – will not receive study data); Susannah J. Tye, Ph.D. (translational neuroscience expert who will provide advice on trial design and planning, meet with other collaborators to evaluate results and planning further studies)

Study Title: Randomized Controlled Trial of Stress Management and Resiliency Training for Depression (SMART-D) vs Treatment as Usual in the treatment of Major Depression

Protocol version number and date: Version 7; June 22, 2021

| **Research Question and Aims** |
| --- |

Background:

A hallmark of major depression is negative attentional and motivational bias, along with anhedonia, rumination, cognitive inefficiencies and social isolation. These deficits impair the ability to exercise adequate cognitive reappraisal and behavioral strategies towards effective problem solving (Chamberlein 2006). The relationship between stress and depression is well established; chronic accumulating effects of stress are associated with risk of developing major depression and are also associated with the maintenance, and recurrence of depression (Kessler 1997, Kendler et al 1999, Brown & Harris 1978, Monroe 1996). Additionally, depression may generate interpersonal stressful conditions that further exacerbate stress and cause chronic or intermittent depression (Hammen 1991, 2003).

Resilience can be broadly defined as the capacity of an individual to resist negative psychological, biological and social consequences of stress. Resilience represents an active and dynamic positive adaptive response to stress. The American Psychological Association defines resiliency as “the process of adapting well in the face of adversity, trauma, tragedy, threats or even significant sources of threat” (APA 2010). Resilience is associated with factors that include: ability to maintain cognitive flexibility and emotional regulation under stress, having previous successful experience of dealing with stress (i.e. stress inoculation), presence of strong social support and role models, spirituality, and living with a sense of purpose and meaning (Southwick & Charney 2012).

A meta-analysis of efficacy of resiliency training programs, focusing primarily on attention control and cognitive reappraisal, in diverse adult populations and people with chronic medical illnesses showed small to moderate effects for improving resilience (pooled SMD 0.37, 95% CI 0.18, 0.57, p = .0002, k =25, I²=41%), reducing stress, and decreasing depression symptom severity (Leppin 2014). There is a paucity of research studies investigating the therapeutic effects of resilience targeted interventions, especially as part of a treatment intervention for syndromal major depression.

Gold standard psychotherapy interventions such as cognitive behavioral therapy focus on depressive symptoms and recovery from a pathological state of a major depressive episode. Unfortunately, the relapse rates’ following recovery from an acute depressive episode is 70% with usual treatment alone (Ma and Teasdale 2004). Mindfulness Based Cognitive Therapy (MBCT) appears to confer benefit by the reduction in relapse and recurrence of major depression when applied following recovery from an acute episode in patients who have recurrent depression (Kuyken 2016). Although resilience as a construct was not studied in this body of literature, the inference drawn from this suggests the possibility that patients may have developed resilience to stress, considering stress is significantly associated with relapse in major depression. The approach of MBCT relies heavily on meditation practices that are hard to teach and learn in most settings where patients with depression typically seek treatment. Hence there is a need to find a resilience therapy that can be easily adopted by the teacher (the provider) and the learner (the patient). Stress Management and Resiliency Training (SMART) is a treatment program that has the ability to be easily integrated into routine clinical practice and shown benefit in several stressed populations (Loprinzi 2011, Werneberg 2018).

This research is focused on finding whether a stress management and resiliency program (SMART) has an augmenting role in the treatment of depression and to prevent relapse after recovery from an acute episode.

Innovation:

Current evidence based interventions for depression focus on depression outcomes. There is paucity of research directly addressing stress, one of the most important etiologic factors in the onset and maintenance of major depression and its recurrence. Baseline resilience moderates the effect of stress on psychopathology. Thus far, studies of resilience interventions have largely focused on participants recruited on the basis of levels of stress rather than a diagnosable mental health condition like major depression. The current research attempts to answer the question whether a stress management and resiliency building strategy, that is built to improve on principles used in existing psychotherapy techniques can improve depression treatment outcomes.

Therefore, the primary aim of this research study is to investigate the efficacy of a stress management and resiliency training program (SMART-D) in patients with major depressive disorder, with a current moderate episode.

Specific Aims:

1. To compare outcomes of depressive symptoms (PHQ-9 and HAM-D) over 6 months following an eight-week program of SMART-D therapy + treatment as usual versus treatment as usual for patients with major depression with a current moderate episode.

Hypothesis: We hypothesize that the intervention will lead to improved depression outcomes by reducing depressive symptoms after recovery from an acute episode and reduce relapse rates over a 6 month period.

1. To compare measures of resilience (Connor Davidson Resiliency Scale) and perceived stress (Perceived Stress Scale) over 6 months following an eight-week program of SMART-D therapy versus treatment as usual for patients with major depression.

Hypothesis: We also hypothesize that the intervention will be associated with improved resilience and decreased perceived stress as secondary outcomes, which are presumably the agents of change with the SMART-D intervention.

1. To assess the influence of genetic polymorphisms of Serotonin Transporter gene (SLC6A4) and Catechol-O-Methyltransferase gene between early adverse experiences, current burden of stress, depression and treatment response to SMART-D therapy.

Hypothesis: We hypothesize that some genetic variations will be associated with greater depression in participants with adverse childhood experiences and subsequently reduced response to SMART-D intervention.

Scientific Approach:

SMART is a resiliency training program created at the Mayo Clinic (Sood 2013). Key components of SMART include identifying emotional signs of stress and recognizing different thinking modes of the brain, such as the default mode and the focused mode. Participants learn attention training and practice finding novelty, using curiosity and kindness to view the physical world, and the relationships within them. The cognitive aspects of training include intentional practice of principles of gratitude, compassion, acceptance, forgiveness, higher meaning and purpose to interpret and reframe day-to-day life experiences.

SMART emphasizes attention training and developing a core mindset to reinterpret life events. The techniques are based on intentional brief daily practices that can be adapted to daily life experiences. In contrast to conventional CBT, the initial target with SMART is not identification of a negative automatic thought to challenge, but a feeling of being stressed. Participants are taught to intentionally reframe or reinterpret the situation by using principles of directed attention such as “kind attention” and principles of gratitude (ex. “thankful for what’s right”), compassion (ex. “kindness to self and others”), Acceptance (ex. “accept what went wrong while working to make things better”), Higher meaning (ex. “what can I learn from this”?) and Forgiveness (ex. “I choose to forgive to let go of my anger”). The emphasis of SMART is on maintaining a resilient mindset using active eudemonic strategies for cognitive reframing and emotional regulation. In contrast to meditation-based strategies of mindfulness-based therapies, SMART emphasizes brief intentional practices. One of the cognitive principles used in SMART are shared with the core principle of Acceptance in Acceptance and Commitment Therapy (ACT), the target of ACT being depressive thought, contact with the present moment and commitment to behavioral change. There are some parallels in the content of SMART with Positive psychotherapy (PP) as designed by Seligman et al., but differ in emphasis and timing of interventions (Seligman et al. 2006). SMART emphasizes the focus on developing resilience as the core target with early attention training and cognitive reframing practices. All core concepts are introduced by the fourth week. SMART therapy can also relatively brief with duration of 4-8 sessions.

In a recent open label feasibility study, patients with major depression were invited to participate in an adjunctive 8-week group therapy of SMART. The primary outcome measure was baseline to endpoint change in resilience as measured by the Connor Davidson Resilience Scale (CD-RISC). Secondary outcome measures included baseline to endpoint change in the Cohen’s Perceived Stress Scale, and depression [Hamilton Rating Scale for Depression (HAM-D) and Patient Health Questionnaire (PHQ-9)]. 23 participants enrolled in the study (mean age = 46±13 years, female =91%). Baseline ratings of mood were of mild to moderate symptom severity (mean HAM-D=14.5 and PHQ-9=12). 74% of participants were study completers (attended ≥ 6 sessions). In an intention-to-treat analysis, at study endpoint, there was a significant improvement in resilience (p = .03), reduction in perceived stress (p =.002) and improvement in depression (HAM-D and PHQ-9- both p<0.001). The study showed that a resilience training program focusing on wellness is feasible for patients who are currently symptomatic with major depression. It was concluded that a larger randomized controlled trial is needed to establish efficacy of this intervention and explore the long-term impact of stress management and resilience training in major depressive disorder (manuscript under review).

During the feasibility study, it was also observed that participants had difficulties in practicing self-kindness and self-compassion. These appeared to be influenced by negative self-esteem and feelings of guilt. Participants also found the topics of acceptance and forgiveness challenging. These challenges may be unique to patients with major depression with their negative cognitive biases. While the emphasis of the course was to develop resilience building strategies towards the present and future, participants showed a tendency to focus on the past and engage in rumination. As a result, modifications to the SMART program were made to create the SMART for depression (SMART-D) for the proposed study. These include specific focus on self-compassion, self-acceptance, self-forgiveness and anger as part of the coursework of modified SMART-D.

| **Study Design and Methods** |
| --- |

**Methods:**

Study Design:

We propose to conduct an open label randomized controlled trial of SMART-D+ treatment as usual versus treatment as usual in a sample of patients in treatment for major depression, with a current moderate episode.Ethics approval will be sought from the Mayo Clinic Institutional Review Board. Study participants will provide voluntary informed consent to enroll into the study before randomization. Study participants who are randomized to the TAU group will be offered the chance to participate in SMART-D groups after completion of the TAU follow-up period of 6 months. Participants who complete the SMART-D groups and the TAU group will be followed up at three and six months after the end of the SMART-D groups.

Participants:

We propose to recruit 80 patients (total) from patients attending the Mayo Clinic Depression Center in Rochester, Mayo Clinic Family Medicine Clinics in Rochester, MN and Kasson, MN and Behavioral Health Clinic at Mayo Clinic Health System, Austin and Albert Lea, MN, and MCHS Red Wing, MN with a diagnosis of major depression, currently exhibiting a moderate episode. Moderate depression will be defined by PHQ-9 scores between 10-19, HAM-D scores ≤23.Participants may also self-refer but would be required to have a primary Family Medicine or Psychiatry provider managing their psychiatric care. Participants will be informed that study participation is voluntary and they can withdraw from the study at any time. Participants will be allowed to continue usual care including medications during the study period as part of treatment as usual care. For participants receiving psychotherapy, the type, duration and frequency of psychotherapy will be tracked. Participants randomized to the SMART-D intervention group will be encouraged to maintain the same medications during the course of the 8 week SMART-D intervention. However, considering that SMART-D intervention is an augmentation strategy to treatment as usual, any medication changes that are made will be noted by the research team. Participants receiving usual care will be allowed to have medication changes or changes in psychotherapy during the entire study duration. These changes will be tracked by the research team.

Procedure:

Potential participants interested in the research study will be invited for a study assessment. Written informed consent will be obtained if participants meet the eligibility criteria as detailed below. Potential participants will also have the option of going over the informed consent form and study assessments via phone or Zoom, and sign the informed consent form electronically. Once enrolled, participants will complete a Structured Clinical Interview for Diagnosis version 4 (SCID) modules A, D, E, F (First et al 2015). The SCID will primarily serve to confirm a diagnosis of Major Depressive Disorder. Additional clinical and demographic characteristics will be obtained during this visit, including age of onset of depression, number of previous lifetime episodes, duration of current or recent depressive episode, current medications and doses, current psychotherapy type and regimen. Baseline clinical measures will be obtained for depression, perceived stress, resilience, quality of life, current burden of stress, adverse childhood experiences, mindfulness, and self-compassion (see Table 1). If the start of SMART-D sessions occurs 4 weeks or greater after randomization, baseline measures will be obtained again. Preferably, at the time of enrollment, 4 ml of whole blood will be collected in a purple top EDTA tube and frozen for storage to be used for a future study, pending funding, of assessing the role of genetic biomarkers of serotonin transporter polymorphism and COMT polymorphism in influencing the relationship between early life and current stress, baseline resilience and depression and subsequent treatment response of SMART-D. For convenience, participants will be allowed to complete the blood draw at any point during their participation in the study. The blood draw can be collected at either Rochester or Austin locations. The samples will not be processed as part of this study.

Follow-up Assessments:

The first follow-up assessment will occur at the end of the SMART-D group therapy for both the comparison arms. The subsequent follow-up will occur at three and six months Post- SMART-D for both arms of the study (see Table 1). Measurements will be carried out, provisioned +2 weeks for either in person, by telephone, or electronically through patient portal [HAM-D (clinician administered scale) will have to be administered by phone or in-person]. Participants will be remunerated $20.00 in the form of a Mayo Clinic check for completing outcome measures at each assessment time point of the study (i.e. Week 1, Week 8, 3 month-Post SMART-D, and 6 months-Post SMART-D visits for a total of $80 per participant.

Symptom Management during enrollment and follow-up:

At study enrollment, participants will complete a PHQ-9 and any elevated score (total score 10 or higher, or any positive response on item 9, a 1, 2 or 3) will be reviewed by a study physician at the enrollment study visit and participants will be assessed for symptoms of depression and risk of suicidality. When the PHQ-9 is completed electronically or my telephone, a study physician will attempt to contact the study participant with an elevated PHQ-9 that same day to assess for symptoms of depression and arrange emergency psychiatric care if needed. Similarly, the HAM-D scores will also be reviewed by the study physician during enrollment and follow-up visits within the same day of completion to assess depressive symptoms and risk of suicidality. Scores above 13 or presence of suicidal ideations will be reviewed by the study physician within the same day. If symptom severity warrants the need for further psychiatric care, prompt clinical measures will be taken by the physician to address the symptoms that include coordinating with the primary care or psychiatric providers involved with the participants care. If a participant needs psychiatric hospitalization during the course of the study, they will have the option of continuing in the research study.

Table 1: Study Assessments*

*participants will have the option of completing all assessments either remotely (phone or Zoom) or in-person

| **Assessment** | **Screening** | | **Week 1 (SMART-D)** | **Week-8**  **(Post-SMART-D)** | | **3 month- Post-SMART-D** | **6 months- Post SMART-D** | |
| --- | --- | --- | --- | --- | --- | --- | --- | --- |
| Informed Consent | **X** | |  |  | |  |  | |
| SCID | **X** | |  |  | |  |  | |
| Connor-Davidson Scale |  | | **X** | **X** | | **X** | **X** | |
| PHQ-9 | **X** | | **X** | **X** | | **X** | **X** | |
| Hamilton Rating Scale | **X** | | **X** | **X** | | **X** | **X** | |
| Cohen Perceived Stress Scale |  | | **X** | **X** | | **X** | **X** | |
| GAD-7 Scale |  | | **X** | **X** | | **X** | **X** | |
|  |  | |  |  | |  |  | |
| LASA- QOL scale |  | | **X** | **X** | | **X** | **X** | |
| Neff’s Self-Compassion Scale |  | | **X** | **X** | | **X** | **X** | |
| Mindful Attention Awareness Scale |  | | **X** | **X** | | **X** | **X** | |
| Current Burden of Stress scale | |  | X |  |  | | |  |
| Adverse Childhood Experiences Questionnaire | |  | X |  |  | | |  |

Randomization and Blinding:

Randomization sequence will be generated using Microsoft Excel 2013 using random block sizes of either three, four, or 5random numbers- depending on how many participants are accrued for one group- by an independent researcher not involved with the study. Allocation sequence will be concealed from the research coordinator assessing participants in sequentially numbered, opaque, sealed and stapled envelopes. Due to the nature of the study design, blinding of participants will not be possible. Outcome assessors will be blind to the group allocation. Study participants will be told not to share their group allocation to the assessors. Statistical analysis will be performed by an independent statistician, who will be blind to the group allocation.

Intervention:

The SMART-D intervention group will comprise of between minimum 4 to maximum 8 research participants per group. The SMART-D intervention will be primarily delivered by 2 therapists with one lead therapist (study PI, Dr. Seshadri) and one co-therapist (Co-I, Ms. Sarah Stinson or Ms. Sherry Chesak) for all groups. The sessions will be provided utilizing a HIPPA compliant real-time audio visual connection via Zoom video through the Patient Portal or telephone calls with the first 4 of 6 sessions occurring weekly and sessions 5 and 6 occurring on week 6 and week 8. In the event of emergencies where the therapist is not available, provisions will be made to have the session delivered by a second therapist, who is also proficient to deliver the intervention (study Co-Investigators – Sherry Chesak or Sarah Stinson). Each session will be 75-90 minute duration, occurring weekly at the same time of the week, except on holidays or inclement weather. The sessions will include a brief introduction and review of homework assignments, followed by a presentation, based on the book “*Mayo Clinic Guide to Stress Free Living*”, and a group discussion of each topic presented. The content of the sessions have been modified in collaboration with the creator of the program based on findings from the feasibility study conducted earlier (see Table 2). To ensure fidelity of the intervention, the study team will select, at random, 1-2 sessions per group to be audio recorded and reviewed by content experts within the research team. SMART-D participants who complete 3 out of 6 contact points will be considered study completers.

Table 2: Structure of Sessions SMART-D

| **Agenda** | **Insight** | **Practices** | |
| --- | --- | --- | --- |
|  |  | Core | Supplementary |
| Week 1 | Science of Stress, The SMART Approach, Gratitude | Morning gratitude | Grateful note, Gratitude jar |
| Week 2 | Mindful Presence and Kindness | Two-minute rule:  Kind Attention | Curious Moments, Kindness to Self,  10 minute relaxation |
| Week 3 | Resilient Mindset- I | Gratitude, Compassion | Dropping one, Finding Inspiration |
| Week 4 | Resilient Mindset-II | Meaning, Acceptance, Forgiveness | Resilient living (Pick One habit) |
| Week 6 | SMART- Integrated approach-I | Integrate core practices | Develop individual ideas |
| Week 8 | SMART- Integrated approach-II |  | Develop individual ideas |

Treatment as Usual:

Treatment as usual will consist of any ongoing medication or psychotherapy based treatments that are currently in place. Participants will be required to be under the supervision of a primary care provider or Psychiatrist or Psychologist for their major depression during their participation in the study.

| **Subject Information** |
| --- |

Target accrual: 80 subjects

Subject population (children, adults, groups): Adults from age 25 - 80

Inclusion Criteria:

- Participants will be enrolled for this study from patients attending the Mayo Clinic Depression Center, Mayo Clinic Family Medicine clinics at Rochester and Kasson, MN, Behavioral Health and Primary Care Clinic at Mayo Clinic Health System, Austin and Albert Lea, MN, and MCHS Red Wing, MN with a diagnosis of major depression with a current moderate episode, with PHQ-9 scores 10-19.
- Participants will be required to be between 25 and 80 years old
- Able to speak English
- Able to provide written informed consent to participate in the study
- Participants must have DSM-V diagnostic confirmation of major depressive disorder (MDD) (American Psychiatric Association 2013).
- Participants will continue taking any prescribed medications from their clinical treatment team.
- Participants with co-morbid secondary diagnoses of persistent depressive disorder and generalized anxiety disorders will be included in the study.
- Participants must consent to audio recording of random group sessions which will be disclosed at the final study session.
- Participants are willing to use the Mayo Clinic Patient Portal for communication purposes during the study.

Exclusion Criteria:

- Participants with bipolar disorder, active psychosis, active suicidal ideations, and active substance abuse meeting criteria for substance use disorders except nicotine, obsessive compulsive disorder, active panic disorder with agoraphobia or other phobic disorder, active posttraumatic stress disorder, active severe personality disorders will be excluded.
- Participants with a severe major depressive episode- HAM-D scores >23.
- Pregnant women – because of time duration of the study.

| **Biospecimens** |
| --- |

Collection of blood samples. When multiple groups are involved copy and paste the appropriate section below for example repeat section b when drawing blood from children and adults with cancer.

1. **From healthy, non-pregnant, adult subjects who weigh at least 110 pounds**. For a minimal risk application, the amount of blood drawn from these subjects may not exceed 550ml in an 8 week period and collection may not occur more frequently than 2 times per week.

Volume per blood draw: __4___ml

Frequency of blood draw (e.g. single draw, time(s) per week, per year, etc.) ___Single Blood draw at study entry________

1. **From other adults and children considering age, weight, and health of subject.** For a minimal risk application, the amount of blood drawn from these subjects may not exceed the lesser of 50 ml or 3 ml per kg in an 8 week period, and collection may not occur more frequently than 2 times per week.

Volume per blood draw: _____ml

Frequency of blood draw (e.g. single draw, time(s) per week, per year, etc.) ___________

Prospective collection of biological specimens other than blood: ______________________________

| **Review of medical records, images, specimens** |
| --- |

Check all that apply (data includes medical records, images, specimens).

Only data that exists before the IRB submission date will be collected.

**Date Range for Specimens and/or Review of Medical Records:**

Examples: *01/01/1999 through 12/31/2015*, or all records through *mm/dd/yyyy*.

Note: The Date Range must include the period for collection of baseline data, as well as follow-up data, if applicable.

The study involves data that exist at the time of IRB submission **and** data that will be generated after IRB submission. Include this activity in the Methods section.

The study will use data that have been collected under another IRB protocol. Include in the Methods section and enter the IRB number from which the research material will be obtained. *When appropriate, note when subjects have provided consent for future use of their data and/or specimens as described in this protocol*.

Enter one IRB number per line, add more lines as needed

Data  Specimens  Data & Specimens ______________________________________

Data  Specimens  Data & Specimens ______________________________________

Data  Specimens  Data & Specimens ______________________________________

| Data Analysis |
| --- |

Outcome Measures:

Outcome measures included measures of depression, perceived stress, resilience and anxiety. Treatment effectiveness will be assessed utilizing the objective ratings of the Hamilton rating scale for depression – 17 item (Hamilton 1960) and self-reported Patient Health Questionaire-9 (Kroenke and Spitzer 2002). The use of two different instruments of depression outcomes comes from a meta-analysis of psychotherapy studies that showed significant differences between clinician-rated and self-report measures of improvement following psychotherapy for depression. This suggests the possibility that different symptoms are suited for subjective or objective ratings (Cuipers 2010). Perceived stress will be measured using the Perceived Stress Scale (PSS), a 10-item self-administered scale that measures the degree to which one’s life is appraised as stressful (Cohen 1983). Anxiety will be measured using the self-reported Generalized Anxiety Questionnaire (Spitzer 2006). Resilience will be assessed by the Connor-Davidson Resilience Scale (CD-RISC) (Connor-Davidson 2003). Internal consistency will be assessed in our sample by measuring omega at baseline and follow-up periods for each group separately. Quality of Life will be measured using a 6 item linear analog self-assessment (LASA), questionnaire. Respondents rate their QOL on a 0–10 numerical analog scale for each domain i.e., mental, emotional, physical, spiritual, social and overall QOL (Singh et al 2014). To measure potential mechanisms of action of SMART therapy, we will measure mindfulness using a 10-item scale, The Mindful Attention Awareness Scale (MAAS), and we will measure self-compassion using a 12-item Self-Compassion Scale- Short form(SCS-SF).

Power Calculation:

Sample size calculation is based on effect sizes obtained from the feasibility study we completed recently. Although we found a within group standardized mean difference >1 for reduction in depressive symptoms in our pilot study, we will assume a moderate standardized mean difference of 0.7 for the RCT, , alpha level =0.05 (two-tailed), power of 80%. Based on these assumptions, we will require a sample of 40 participants in each group providing allowance for 25% drop-out rate to detect statistically significant differences in depressive symptoms between the 2 groups.

Statistical Analysis:

The SMART-D and TAU groups will be compared on baseline demographic and clinical characteristics. Primary analysis will apply Intention-to-treat analysis to compare treatment outcomes of depression, with participants analyzed based on their initial randomization, at post-SMART-D intervention, at the 3 month and 6 month time post intervention time points. Analysis of the primary outcome (depression) and secondary outcomes (perceived stress, resilience) will be carried out using linear mixed effects regression with results presented as mean differences (and 95% CIs). Secondary analysis will examine the relationship of stress as a predictor of depression and depression as a predictor of stress. For each outcome, a single mixed effects model will be fitted incorporating baseline, post-intervention, 3- and 6-month data using random effects to allow for the repeated measures within an individual, and clustering of individuals within each treatment site. Separate parameters will be estimated for group differences at 3- and 6-months post-intervention; scores at baseline will be added as a covariate. Differences in the outcomes of SMART-D and TAU groups will be assessed using linear mixed models, with individuals clustered within each treatment site and assessment times clustered within individuals. We will run an unadjusted model and compare this to an adjusted model, with the unadjusted model as our primary analysis, to evaluate robustness of these findings. The adjusted model will include the following as covariates: gender, age, education, marital status, medication use (obtained from interview and chart records), number of previous depressive episodes, baseline anxiety (measured by GAD) and severity of recent depressive episode. The participant’s site and individual participant will be modelled as random effects and the remaining variables will be handled as fixed effects. Conditional maximum likelihood will be used to fit the mixed model to account for the missing data. This approach makes an assumption that data are missing at random. Sensitivity analysis in the form of pattern mixture models will be used to explore the departure from this assumption. We will also perform a per-protocol analysis as a supplementary analysis. Significance will be tested at p < .05 for the primary analysis. False Discovery Rate method will be used to make adjustments for Type I error inflation for secondary outcomes.

References

1. S. Ma, J.D. Teasdale Mindfulness-based cognitive therapy for depression: replication and exploration of differential relapse prevention effects. J. Consult. Clin. Psychol., 72 (2004), pp. 31-40

2. Brown GW, Harris TO. Social Origins of Depression. New York, NY, The Free Press, 1978

3. Kendler KS, Karkowski LM, Prescott CA. Causal relationship between stressful life events and the onset of major depression. Am J Psychiatry 1999;156:837-841

4. Kessler RC. The effects of stressful life events on depression. Annu Rev Psychol 1997;48:191-214

5. Monroe SM, Roberts JE, Kupfer DJ, et al. Life stress and treatment course of recurrent depression: II. Postrecovery associations with attrition, symptom course, and recurrence over 3 years. J Abnorm Psychol 1996;105:313-328

6. American Psychological Association. The Road to Resilience. Washington, DC, American Psychological Association, 2010

7. Leppin AL, Bora PR, Tilburt JC, et al. The efficacy of resiliency training programs: a systematic review and meta-analysis of randomized trials. PLoS One 2014;9:e111420

8. Chamberlain SR, Sahakian BJ. The neuropsychology of mood disorders. Curr Psychiatry Rep 2006;8:458-463

9. Hammen C. Generation of stress in the course of unipolar depression. J Abnorm Psychol 1991;100:555-561

10. Hammen C. Interpersonal stress and depression in women. J Affect Disord 2003;74:49-57

11. Southwick SM, Charney DS. The science of resilience: implications for the prevention and treatment of depression. Science 2012;338:79-82

12. Loprinzi CE, Prasad K, Schroeder DR, et al. Stress Management and Resilience Training (SMART) program to decrease stress and enhance resilience among breast cancer survivors: a pilot randomized clinical trial. Clin Breast Cancer 2011;11:364-368

13. Werneburg BL, Jenkins SM, Friend JL, et al. Improving Resiliency in Healthcare Employees. Am J Health Behav 2018;42:39-50

14. Sood A. The Mayo Clinic Guide to Stress-Free Living. Boston, MA, Da Capo Press, 2013

15. Seligman MEP, Rashid T, Parks AC. Positive psychotherapy. Am Psychol 2006;61:774-788

16. Hamilton M. A rating scale for depression. J Neurol Neurosurg Psychiatry 1960;23:56-62

17. Kroenke K, Spitzer RL. The PHQ-9: A New Depression Diagnostic and Severity Measure. Psychiatr Ann 2002;32:509-521

18. Spitzer RL, Kroenke K, Williams JB, et al. A brief measure for assessing generalized anxiety disorder: the GAD-7. Arch Intern Med 2006;166:1092-1097

19. Cohen S, Kamarck T, Mermelstein R. A global measure of perceived stress. J Health Soc Behav 1983;24:385-396

20. Connor KM, Davidson JR. Development of a new resilience scale: the Connor-Davidson Resilience Scale (CD-RISC). Depress Anxiety 2003;18:76-82

21. Singh JA, Satele D, Pattabasavaiah S, Buckner JC, Sloan JA. Normative data and clinically significant effect sizes for single-item numerical linear analogue self-assessment (LASA) scales. Health and Quality of Life Outcomes 2014;12, Article number: 187

22. First MB, Williams JBW, Karg RS, et al. Structured Clinical Interview for DSM-5—Research Version (SCID-5 for DSM-5, Research Version; SCID-5-RV). Arlington, VA, American Psychiatric Association, 2015

23 MacKillop J, Anderson EJ. Further Psychometric Validation of the Mindful Attention Awareness Scale (Maas). Journal of Psychopathology and Behavioral Assesment. 2007;29(4):289-293.

24 Raes F, Pommier E, Neff KD, Van Gucht D. Construction and Factorial Validation of a Short Form of the Self-Compassion Scale. Clin Psychol Psychother. May-Jun 2011;18(3):250-255.
